# Circ-0006332 stimulates cardiomyocyte pyroptosis via the miR-143/TLR2 axis to promote doxorubicin-induced cardiac damage

**DOI:** 10.1080/15592294.2024.2380145

**Published:** 2024-07-17

**Authors:** Ping Zhang, Yuanyuan Liu, Yuliang Zhan, Pengtao Zou, Xinyong Cai, Yanmei Chen, Liang Shao

**Affiliations:** a Department of Neurology, Jiangxi Provincial People’s Hospital, The First Hospital Affiliated to Nanchang Medical College, Nanchang, Jiangxi, China; b Department of Cardiology, Jiangxi Provincial People’s Hospital, The First Affiliated Hospital to Nanchang Medical College, Nanchang, Jiangxi, China

**Keywords:** microRNA-143, circular RNA-0006332, TLR2, pyroptosis, doxorubicin, heart failure

## Abstract

Doxorubicin (DOX)-mediated cardiotoxicity can impair the clinical efficacy of chemotherapy, leading to heart failure (HF). Given the importance of circRNAs and miRNAs in HF, this paper intended to delineate the mechanism of the circular RNA 0006332 (circ -0,006,332)/microRNA (miR)-143/Toll-like receptor 2 (TLR2) axis in doxorubicin (DOX)-induced HF. The binding of miR-143 to circ -0,006,332 and TLR2 was assessed with the dual-luciferase assay, and the binding between miR-143 and circ -0,006,332 was determined with FISH, RIP, and RNA pull-down assays. miR-143 and/or circ -0,006,332 were overexpressed in rats and cardiomyocytes, followed by DOX treatment. In cardiomyocytes, miR-143 and TLR2 expression, cell viability, LDH release, ATP contents, and levels of IL-1β, IL-18, TNF-α, and pyroptosis-related molecules were examined. In rats, cardiac function, serum levels of cardiac enzymes, apoptosis, myocardial fibrosis, and levels of IL-1β, IL-18, TNF-α, TLR2, and pyroptosis-related molecules were detected. miR-143 diminished TLR2 expression by binding to TLR2, and circ -0,006,332 bound to miR-143 to downregulate miR-143 expression. miR-143 expression was reduced and TLR2 expression was augmented in DOX-induced cardiomyocytes. miR-143 inhibited DOX-induced cytotoxicity by suppressing pyroptosis in H9C2 cardiomyocytes. In DOX-induced rats, miR-143 reduced cardiac dysfunction, myocardial apoptosis, myocardial fibrosis, TLR2 levels, and pyroptosis. Furthermore, overexpression of circ -0,006,332 blocked these effects of miR-143 on DOX-induced cardiomyocytes and rats. Circ -0,006,332 stimulates cardiomyocyte pyroptosis by downregulating miR-143 and upregulating TLR2, thus promoting DOX-induced cardiac injury.

## Introduction

Doxorubicin (DOX) is an effective anthracycline used in several diseases, but its application is restricted by severe adverse effects, especially cardiotoxicity [1]. As reported, the cardiotoxicity of DOX affects approximately 30% of patients within 5 years after chemotherapy and leaves permanent cardiac damage [2]. Heart failure (HF) is the primary non-cancer contributor to death in patients receiving DOX treatment [3]. HF patients may experience a number of clinical symptoms, including breathing difficulty, lung congestion, and peripheral oedema, as well as nausea, loss of appetite, and fatigue in daily life [4]. Of note, many molecular mechanisms, including inflammation, fibrosis, and apoptosis, have been revealed to explain DOX-induced cardiotoxicity (DIC) in past decades [5].

Inflammation and fibrosis are intertwined mechanisms in HF [6]. Inflammatory cytokines modulate phenotype and function of all cardiomyocytes, hinder contractile function, stimulate microvascular dysfunction, chronic fibrosis, and adverse cardiac remodelling [7]. During inflammation of the heart, immune cells infiltrate the cardiac tissue and monitor responses that damage the tissue, while cardiac fibrosis manifests as an increased amount and disturbed composition of extracellular matrix proteins [8]. In recent years, emerging regulated cell death pathways, including pyroptosis, have been implicated in DIC [9]. Since it was first discovered in 2001, pyroptosis has been attracting considerable interest for its association with innate immunity and disease [10]. A prior study revealed that doxorubicin-induced pyroptosis elevated NLR family pyrin domain-containing 3 (NLRP3) levels, activated caspase-1, cleaved gasdermin D N-terminus (GSDMD-N), and released Interleukin (IL)-1β and IL-18 in cardiomyocytes [11]. Another study indicated that inhibition of pyroptosis-related molecules might be a strategy to prevent DIC [12].

Numerous studies have confirmed the importance of Toll-like receptors (TLRs) in DIC [13]. Moreover, Hwang et al. observed that DOX enhanced TLR2 expression and stimulated pyroptosis in H9c2 cardiomyocytes and that TLR2 downregulation might be implicated in the attenuation of DOX-induced cardiomyocyte pyroptosis [14]. Therefore, further analysis of the regulatory mechanism of TLR2 May more effectively reduce the cardiotoxicity caused by radiotherapy and chemotherapy. MicroRNAs (miRNAs/miRs) have various biological functions in the heart and are stable and easily detected, which therefore have the potential to be biomarkers and targets for DIC [15]. Among miRNAs, miR-143 has been verified to target the 3’-untranslated region of TLR2 in various diseases, such as Propionibacterium acnes-mediated inflammation in skin and colorectal carcinoma [16,17]. A decrease in miR-143 expression is significant in patients with severe pulmonary hypertension which can result in HF [18]. miR-143-3p is expressed at significantly lower levels in the heart of streptozocin-induced diabetic rats than non-diabetic controls [19].

As a unique class of the noncoding RNA family, circular RNAs (circRNAs) are ubiquitous in mammalian cells and tissues, showing complex regulatory roles in cardiovascular diseases [20]. For example, circ_0001312 relieves the DIC via the miR-409-3p/high-mobility group box 1(HMGB1) axis [21]. Circ-LTBP1 enhances DOX-induced inflammation and apoptosis in AC16 cells via the miR-107/ADCY1 axis [22]. Mounting evidence has reported the ability of circRNAs as miRNA sponges to mediate RNA expression and functions, which have gained great attention in various research fields [23]. Of note, a previous study manifested that circ_0006332, produced from the splicing of MYBL2, sponged miR-143 to regulate bladder cancer cells [24].

Given the above information, we reasonably hypothesize that circ_0006332 May modulate TLR2 through miR-143 to be involved in DIC. On this basis, this study probed the regulatory roles of the circ_0006332/miR-143/TLR2 axis in cardiomyocyte pyroptosis during DOX-induced HF.

## Methods

### Ethics statement

This study was ratified by the Animal Ethical Committee of our hospital. All animal experiments complied with the Guide for the Care and Use of Laboratory Animals (NIH, 76 FR 91). All efforts were made to minimize rat number and their pain.

### Cell culture and treatment

H9C2 cells (Cell Resource Center, Peking Union Medical College, Beijing, China) were cultured with Dulbecco’s modified Eagle’s medium encompassing 10% foetal bovine serum and 1% penicillin/streptomycin in a 37°C incubator containing saturated humidity and 5% CO_2_. Subsequent to transfection for 12–16 h and state recovery, H9C2 cells were stimulated with 1 μg/mL of DOX [25] for 24 h, and the cells and cell supernatant were collected for subsequent assays.

### Cell transfection

To assess the action of miR-143 on DIC, the following groups were set up at the cellular level: Vehicle + mimic negative control (NC), DOX + mimic NC, and DOX + miR-143 mimic groups. To elucidate whether circ -0,006,332 mediates DIC via miR-143, we designed the following groups, including Vehicle+mimic NC + overexpression (oe)-NC, DOX + mimic NC + oe-NC, DOX + miR-143 mimic + oe-NC, and DOX+ miR-143 mimic + oe-circ -0,006,332 groups. GenePharma (Shanghai, China) was entrusted to design and synthesize the above sequences. After cell density was adjusted, cells were seeded in the culture plate to ensure 70%-80% confluence on the second day of transfection. Transfection was performed with Lipofectamine 3000. Briefly, 250 μL of Opti-MEM medium (Gibco, Grand Island, NY, USA) was adopted to dilute target plasmids (4 μg) and Lipofectamine 3000 (10 μL), respectively. The two liquids were mixed evenly after standing for 5 min and added to the wells, and the medium was renewed with a fresh medium after 4–6 h of transfection. After 48 h, cells were removed or treated for subsequent experimentation after state recovery [26].

### Western blotting

Tissue homogenates or cells were fully lysed on ice with radio-immunoprecipitation assay (RIPA) Protein Lysis Solution (P0013B, Beyotime, Shanghai, China), and protein concentration was estimated with a bicinchoninic acid kit (P0012S, Beyotime). After that, 10 μL loading buffer was boiled at 95°C for 10 min before sample loading for electrophoresis in 100 V lanes. Next, the proteins were placed on nitrocellulose membranes (30 mA, 120 min), sealed with a sealing solution [5% bovine serum albumin/Tris-buffered saline-tween (TBST)] for 60 min, and incubated overnight with the following primary antibodies: TLR2 (ab209217, 1:1000, Abcam, Cambridge, UK), NLRP3 (ab263899, 1:1000, Abcam), ASC (ab307560, 1:1000, Abcam), cleave-caspase-1 (#89332, Cell Signaling Technology, Beverly, MA, USA), GSDMD-N (ab239377, 1:1000, Abcam), HMGB1 (ab18256, 1:1,000, Abcam), and GAPDH (ab9484, 1:5000, Abcam) at 4°C. Next, the membranes were washed in 1× TBST solution on the shaker (5 min × 3) and probed with goat anti-rabbit immunoglobulin G (IgG) H&L (horseradish peroxidase) secondary antibody (ab6721, 1:5000, Abcam) for 2 h, followed by TBST washing (20 min × 3). Luminescence reactions were performed with an enhanced chemiluminescence kit to observe protein blotting. The greyscale values of bands were analysed with Image J software.

### Reverse transcription-quantitative polymerase chain reaction (RT-qPCR)

Collected cell or tissue samples were utilized for total RNA extraction, which was conducted with TRIzol (15596026, Invitrogen, Car, USA). RNA was reverse-transcribed into cDNA with a RT kit (RR047A, Takara, Tokyo, Japan) at 37°C for 15 min and at 85°C for 5 s, while the miRNA was reverse-transcribed into cDNA with the miRNA RT Kit (4366596, Thermo Fisher Scientific, Carlsbad, CA, USA). RT-qPCR was performed in a real-time fluorescent qPCR instrument (ABI7500, ABI, Foster City, CA, USA) with the SYBR Premix EX Taq kit (RR420A, Takara). The 20 μL reaction system included 9 μL SYBR Mix, 0.5 μL forward primer, 0.5 μL reverse primer, 2 μL cDNA, and 8 μL RNase Free dH_2_O. The reaction conditions were described below: 95°C for 10 min, 95°C for 15 s, and 60°C for 1 min, for 40 cycles. Primers (Table 1) were synthesized by Sangon Biotech (Shanghai, China). With GAPDH as the internal reference for normalizing mRNAs and U6 for miR-143, the 2^−ΔΔCt^ method was employed to calculate the relative expression [27].

**Table 1.** Primer sequences for RT-qPCR.

Gene	Sequence
miR-143	F: 5’−CTGGCGTTGA GATGAAGCAC3’
	R: 5’-CAGAGCAGGGTCCGAGGTA-3’
circ -0,006,332	F: 5’−ACCGGGACAAGACACCCC3’
	R: 5’-GCAGCTGCACTAGGCTGT-3’
TLR2	F: 5’- CCCAAGCACACTCACTCAACT-3’
	R: 5’-GGGTTCGTGTGAGTGAGTTGA −3’
GAPDH	F: 5’-TCTCTGCTCCTCCCTGTTCT-3’
	R: 5’-ATCCGTTCACACCGACCTTC-3’
U6	F: 5’-AAAGCAAATCATCGGACGACC-3’
	R: 5’-GTACAACACATTGTTTCCTCGGA-3’

### Cell counting kit-8 (CCK-8) assay

After trypsinization, the cells were resuspended, and the cell density was adjusted to 5 × 10^3^ cells/mL, followed by overnight cell culture in 96-well plates at 100 μL/well. Cell viability was tested with the CCK-8 kit (C0037, Beyotime). For each assay, 10 μL CCK-8 assay solution was added for 4-h cell incubation. The absorbance at 450 nm was measured with a microplate marker.

### Lactate dehydrogenase (LDH) release assay

The LDH Cytotoxicity Assay kit (C0016, Beyotime) was used. After centrifugation at 400 ×g for 5 min and collection of the cell supernatant, 120 μL cell supernatants were put in 96-well plates for 30-min incubation in dark. The absorbance at 490 nm was measured. The wavelength ≥600 nm served as a reference.

### Adenosine triphosphate (ATP) measurement

The ATP assay kit (No. S0026, Beyotime) was employed for this assay. About 2 × 10^4^ cells and 200 μL lysis solution were added to each well for adequate cell lysis at 4°C. The ATP assay working solution was made by diluting ATP assay reagents at 1:9 with the ATP assay reagent dilution solution. Then, 20 μL of samples or standards and 100 μL ATP assay working solution were added to each well, mixed well, and then assayed by chemiluminescence.

### Fluorescence in situ hybridization (FISH) assay

The FISH assay was applied for determining the subcellular localization of miR-143 and circ -0,006,332 in cardiomyocytes as per the protocols of the FISH Kit for RNA (R0306S, Beyotime). Briefly, cardiomyocytes were seeded in 6-well plates. Subsequent to fixation, permeabilization, and pre-hybridization, cells at 80% confluence were immersed with hybridization solution containing probes, sealed, and placed on a shaker in dark at 45–65°C for overnight hybridization. After nuclei staining with 4,’6-diamidino-2-phenylindole, the cells were sealed with anti-fluorescent quenching agents, followed by photography under a fluorescence microscope (Olympus, Tokyo, Japan) [28].

### Dual-luciferase assay

The wild-type (WT) and mutant (MUT) TLR2 or circ -0,006,332 was respectively cloned into luciferase reporter gene vectors, which were co-transfected with miR-143 mimic or mimic NC into cardiomyocytes. Cells were lysed 48 h after transfection and assayed for luciferase activity with a luciferase assay kit (RG005, Beyotime) and a dual-luciferase analysis system (Promega, Madison, WI). The plasmids used above were purchased from GenePharma [29].

### RNA immunoprecipitation (RIP)

According to the manuals of the RIP kit (Millipore, Bedford, MA, USA), cells were first washed once in pre-cooled phosphate-buffered saline (PBS) and lysed with RIPA lysis solution (P0013B) on ice for 5 min, followed by centrifugation at 14,000 g and 4°C for 10 min to harvest the supernatant and incubation of cell extracts with antibodies for co-precipitation. In each co-precipitation system, the magnetic beads (50 μL) were resuspended in RIP Wash Buffer (100 μL) and incubated with 1 μg Argonaute2 (Ago2) rabbit monoclonal antibody (ab186733, 1:30, Abcam) for binding, with IgG (ab172730, 1:100, Abcam) as the NC. The bead-antibody complex was resuspended in RIP Wash Buffer (900 μL) after washing and incubated overnight at 4°C with cell extracts (100 μL). Thereafter, samples were put on the magnetic base to get the bead-protein complexes. Following detachment with proteinase K, RNA was extracted for PCR.

### RNA pull-down assay

Based on the manuals of the Pierce^TM^ Magnetic RNA-Protein Pull-Down kit (Millipore), the biotinylated miR-143 probe or control probe (Geneseed, Guangzhou, China) was incubated with cell lysate at 25°C for 2 h. The complexes were captured with streptavidin-labelled immunomagnetic beads and incubated with proteinase K-contained buffer for 1 h. Eluted complexes were measured with RT-qPCR [30].

### Animal experiments

Male Sprague-Dawley rats (aged 8–10 weeks; Hunan SJA Laboratory Animal Center, Changsha, Hunan, China) were housed and bred in a sterile environment with the following condition: 12-h light-dark cycles, 24 ± 1°C, and 50–60% humidity, at the animal centre and fed a standard diet and water. To ascertain the effect of miR-143 on DIC, rats were randomized into Vehicle+agomir NC, DOX + agomir NC, and DOX + miR-143 agomir groups. A rat model of DIC was established by intraperitoneal injection of DOX at 2.5 mg/kg, and rats in the Vehicle group were injected with the corresponding volume of 0.9% NaCl once a week for 6 weeks [31]. Weekly tail vein injection of 25 μg miR-143 agomir or agomir NC was given on days 2 and 4 after DOX administration, twice a week for 6 weeks [32]. (see Figure 7)

Next, to investigate whether circ -0,006,332 mediates DIC via miR-143, rats were randomly allocated into Vehicle + agomir NC + oe-NC, DOX + agomir NC + oe-NC, DOX + miR-143 agomir + oe-NC, and DOX + miR-143 agomir + oe-circ -0,006,332 groups. Rats were given 25 μg oe-circ -0,006,332 or oe-NC adeno-associated viruses via tail vein according to the previous method [33–35]. Weekly administration of DOX at 2.5 mg/kg via intraperitoneal injection was initiated on day 3 after virus injection and lasted for 6 weeks. Weekly tail vein injection of miR-143 agomir or agomir NC was given on days 2 and 4 after DOX administration, twice a week for 6 weeks. Two weeks after the last DOX administration, rats were anesthetized via isoflurane inhalation, and blood was harvested from the heart, allowed to stand at 4°C for 30 min, and centrifuged to collect serum. The hearts were retained for subsequent testing and analysis. The miR-143 agomir sequence and oe-circ -0,006,332 adeno-associated virus sequence used above were designed and synthesized by GenePharma.

### Echocardiography

As previously described [36], rats were anesthetized and fixed, followed by echocardiography with an ultrasound machine (M5 Vet, Mindray, Shenzhen, China) to evaluate cardiac function. Thereafter, left ventricular (LV) ejection fraction (LVEF), LV shortening fraction (LVFS), LV end-systolic diameter (LVEDS), and LV end-diastolic diameter (LVEDD) were measured and calculated with the attached software.

### Enzyme-linked immunosorbent assay (ELISA)

IL-1β, IL-18, and tumour necrosis factor-α (TNF-α) in rat serum or cell supernatants were measured with ELISA (Elabscience, Wuhan, Hubei, China). Cell supernatants were collected and standard solutions and enzyme couplers were prepared. The used antigen was diluted to an appropriate concentration with the coating diluent, and antigen diluent was added to wells (100 μL/well) and placed at 37°C for 4 h. The liquid in the wells was discarded. The wells were sealed with 5% calf serum at 37°C for 40 min and washed thrice (3 min each). Afterwards, the liquid in the wells was drained, and the samples were dried with absorbent paper. Then, each well was supplemented with 100 μL diluted samples, incubated for 40–60 min, and washed thrice. Each well was set up with replicate wells. After that, 100 μL enzyme-labelled antibody was added to each well for 30–60 min of incubation, and the wells were washed as before. Subsequent to the addition of 100 μL reaction substrates, the plate was placed at 37°C for 3–5 min in dark. The colour development was terminated by adding 50 μL termination solution into each well, and finally, the absorbance at 450 nm was read on the microplate reader within 20 min.

### Detection of myocardial injury markers

The levels of serum cardiac enzymes, including creatine kinase isoenzyme MB (CK-MB), cardiac troponin T (cTnT), and LDH, are markers of myocardial injury. The levels of CK-MB (H197-1-1, Nanjing Jiancheng Biotechnology Institute, Nanjing, China), LDH (A020-2-2, Nanjing Jiancheng), and cTnT (Milliplex Company, Darmstadt, Germany) in rat serum were measured as instructed in the manuals of corresponding kits.

### Masson staining

After being rinsed with PBS, the heart tissues were fixed in 4% paraformaldehyde, embedded in paraffin, and sliced at 5 μm. As per the protocols of the Masson staining kit (60532ES58, YEASEN, Shanghai, China), the slices were dewaxed, hydrated, stained with haematoxylin, Ponceau S dye, and toluidine blue in turn, followed by xylene treatment and sealing. Finally, they were observed and photographed under a general light microscope (Olympus), and the area of fibrosis in the heart tissue was analysed and evaluated with Image J.

### TUNEL staining

After PBS rinsing, the heart tissues were fixed with 4% paraformaldehyde, paraffin-embedded, and sliced at 5 μm. Based on the protocols of the TUNEL staining kit (G1507, Servicebio, Wuhan, China), the slices were dewaxed, hydrated, and incubated with proteinase K for 20 min. After 1-h incubation with TUNEL reaction solutions, the slices were stained with 3,3'-diaminobenzidine solution, washed thoroughly with PBS, and finally photographed under a general light microscope. Images were analysed and evaluated with Image J. Cells positive for TUNEL staining were those with brown staining in the nucleus.

### Statistical analysis

GraphPad Prism 8.0.1 (GraphPad Software, San Diego, CA, USA) was applied for processing all data. Measurement data were depicted in the form of mean ± standard deviation and conformed to normal distribution and homogeneity of variance. Each set of cell experiments was repeated three times. The *t*-test and one-way analysis of variance were respectively utilized for comparisons between two groups and among multiple groups, with Tukey’s test for post hoc multiple comparisons. The normal distribution test was performed by Shapiro-Wik test. The data that do not conform to the normal distribution were expressed in the form of M (P_25_, P_75_) and tested with non-parameters. *p* < 0.05 represented statistical significance.

## Results

### miR-143 represses DOX-induced cardiomyocyte injury and pyroptosis in vitro

**Figure 1.**
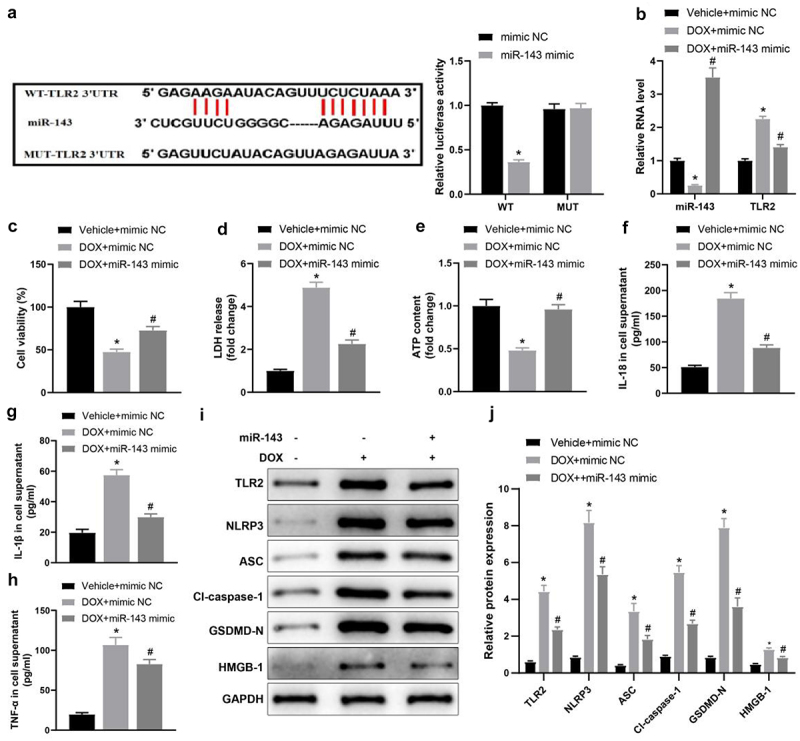
miR-143 reduces injury and pyroptosis in DOX-treated cardiomyocytes. a: dual-luciferase assay to analyse the targeting relationship between miR-143 and TLR2; b: RT-qPCR to detect the levels of miR-143 and TLR2; c: CCK-8 assay to detect cell viability; d: the LDH release kit to detect LDH release; e: ATP production; f: ELISA to detect IL-18 levels in cell supernatant; g: ELISA to detect IL-1β levels in cell supernatant; h: ELISA to detect TNF-α levels in cell supernatant; i-j: Western blotting to detect protein levels of TLR2 and pyroptosis-related molecules. In panels b-h and j, *p < 0.05 compared with the vehicle + mimic NC group, #*P* < 0.05 compared with the DOX + mimic NC group. Measurement data were expressed as mean ± standard deviation. The independent sample *t*-test was used for comparisons between two groups, and one-way ANOVA with Tukey’s post hoc test was used for comparisons among multiple groups. The experiments were repeated three times.

Reportedly, TLR2 is a target gene of miR-143 and upregulated in mice with DOX-stimulated chronic cardiomyopathy [37,38]. Accordingly, the dual-luciferase assay was carried out to probe whether miR-143 targeted TLR2 in DIC. The results revealed that the luminescence activity was reduced by miR-143 mimic (Figure 1(a), *p* < 0.05), whereas the luminescence activity was not obviously changed by miR-143 mimic when the binding site of miR-143 to TLR2 was mutated. This result demonstrated that miR-143 could bind to TLR2 in H9C2 cardiomyocytes, indicating that miR-143 could play a regulatory role in DIC by acting on TLR2.

Cells were transduced with miR-143 mimic and treated with DOX to evaluate the impacts of miR-143 on DIC. RT-qPCR revealed lower miR-143 expression but higher TLR2 expression in the DOX + mimic NC group (vs. the Vehicle + mimic NC group), which was opposite in the DOX + miR-143 mimic group (vs. the DOX + mimic NC group) (Figure 1(b), *p* < 0.05).

The CCK-8 assay indicated that cell viability was notably lower in the DOX + mimic NC group (vs. the Vehicle + mimic NC group) but strikingly higher in the DOX + miR-143 mimic group (vs. the DOX + mimic NC group) (Figure 1(c), *p* < 0.05). Moreover, LDH release in cardiomyocyte supernatants was enhanced and intracellular ATP contents were diminished in the DOX+ mimic NC group (vs. the Vehicle + mimic NC group), which was reverse in the DOX + miR-143 mimic group (vs. the DOX + mimic NC group) (Figure 1d,e, *p* < 0.05). ELISA results unveiled that IL-18, IL-1β, and TNF-α levels in cell supernatants were elevated in the DOX + mimic NC group (vs. the Vehicle + mimic NC group) but greatly reduced in the DOX + miR-143 mimic group (vs. the DOX + mimic NC group) (Figure 1f-h, *p* < 0.05).

Further, data from Western blotting manifested that relative to the Vehicle+ mimic NC group, the protein levels of TLR2 and pyroptosis-related molecules (NLRP3, ASC, cleave-caspase-1, GSDMD-N, and HMGB1) were substantially augmented in the DOX+ mimic NC group. In contrast to the DOX + mimic NC group, but markedly decreased in the DOX + miR-143 mimic group (Figure 1i-j, *p* < 0.05). Altogether, miR-143 overexpression repressed DIC by declining cardiomyocyte pyroptosis.

### miR-143 improves DOX-induced myocardial injury in rats

**Figure 2.**
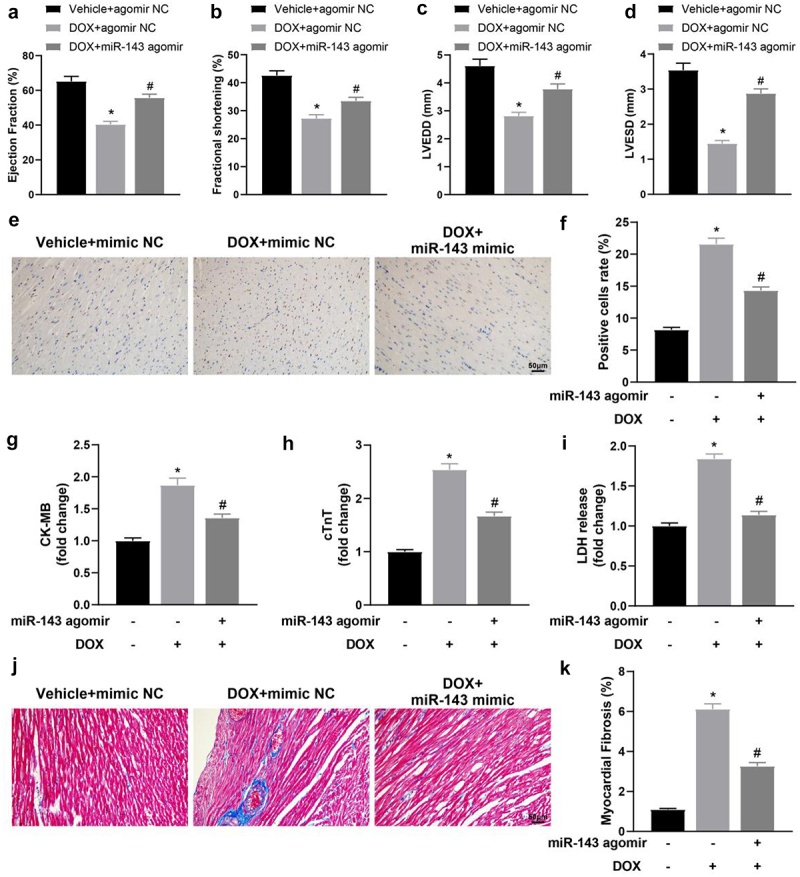
miR-143 improves myocardial injury in DOX-induced (2.5 mg/kg) rats (*n* = 5). a-d: quantitative analysis of echocardiographic detection of cardiac function (LVEF, LVFS, LVEDD, and LVESD); e-f: TUNEL staining to detect apoptosis in myocardial tissues; g-i: ELISA to detect levels of CK-MB, cTnT, and LDH in myocardial tissues; j-k: Masson staining to observe myocardial tissue fibrosis, scale bar = 50 μm; *p < 0.05 compared with the vehicle + agomir NC group, #*P* < 0.05 compared with the DOX + agomir NC group. Values in the figure were measurement data and expressed as mean ± standard deviation. One-way ANOVA with Tukey’s post hoc test (a-d, f-i, k) was used for comparisons among multiple groups.

Firstly, echocardiography showed that the values of LVEF, LVFS, LVEDD, and LVESD were significantly decreased in the DOX + agomir NC group (vs. the Vehicle + agomir NC group) but were enhanced in the DOX + miR-143 agomir group (vs. the DOX + agomir NC group) (Figure 2a-d, *p* < 0.05). The above results suggest that miR-143 can reduce DOX-induced cardiac dysfunction. TUNEL staining results showed that TUNEL-positive cells in rat myocardial tissues were prominently higher in the DOX + agomir NC group (vs. the Vehicle + agomir NC group) but lower in the DOX + miR-143 agomir group (vs. the DOX + agomir NC group) (Figure 2e,f, *p* < 0.05). Serum levels of CK-MB, cTnT and LDH were greatly higher in the DOX + agomir NC group (vs. the Vehicle + agomir NC group) but decreased in the DOX + miR-143 agomir group (vs. the DOX + agomir NC group) (Figure 2g-i, *p* < 0.05). Additionally, Masson staining exhibited that compared with the Vehicle + agomir NC group, the DOX + agomir NC group had markedly enhanced myocardial tissue fibrosis, which was contrary in the DOX + miR-143 agomir group (vs. the DOX + agomir NC group) (Figure 2j,k, *p* < 0.05).

Briefly, miR-143 can reduce DOX-induced myocardial injury in rats by decreasing DOX-induced cell death, reducing myocardial fibrosis, and improving cardiac insufficiency.

### miR-143 prevents DOX-induced pyroptosis in rats

**Figure 3.**
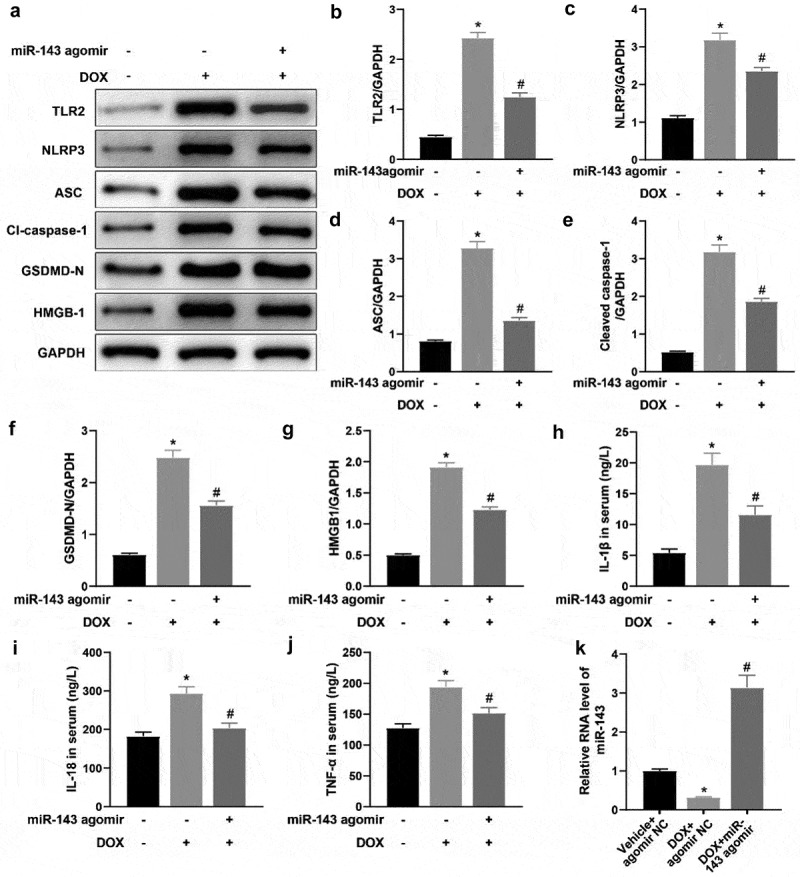
miR-143 prevents DOX-induced cardiomyocyte pyroptosis in vivo. a-g: Western blotting to detect levels of TLR2 protein and pyroptosis-related proteins (ASC, cleave-caspase-1, GSDMD-N, NLRP3, and HMGB1) in cardiac tissues; h-j: ELISA to detect IL-1β, IL-18, and TNF-α in rat serum; k: RT-qPCR to detect miR-143 expression in myocardial tissues; *p < 0.05 compared with the vehicle + agomir NC group, #*P* < 0.05 compared with the DOX + agomir NC group. Values in the figure were measurement data and expressed as mean ± standard deviation. One-way ANOVA and Tukey’s post hoc test were used for comparison among multiple groups (b-k).

To delineate the specific molecular mechanism of miR-143 ameliorating DOX-induced myocardial injury, rat heart tissues were collected to examine protein levels of TLR2 and pyroptosis-related molecules in heart tissues. In contrast to the Vehicle + agomir NC group, these protein levels were greatly augmented in the DOX + agomir NC group, whilst the DOX + miR-143 agomir group showed reverse trends versus the DOX + agomir NC group (Figure 3a-g, *p* < 0.05). Serum levels of IL-1β, IL-18, and TNF-α in the DOX + agomir NC group were notably increased as compared to those in the Vehicle + agomir NC group. Conversely, the levels of these factors decreased in the DOX + miR-143 agomir group in comparison to the DOX + agomir NC group (Figure 3h–j, *p* < 0.05). RT-qPCR results demonstrated lower miR-143 expression in heart tissue in the DOX + agomir NC group (vs. the Vehicle + agomir NC group) but higher expression in the DOX + miR-143 agomir group (vs. the DOX + agomir NC group) (Figure 3k, *p* < 0.05). Conclusively, miR-143 upregulation may suppress DIC in rats by reducing TLR2 expression and the pyroptosis pathway.

### Circ -0,006,332 overexpression aggravates DOX-stimulated cardiomyocyte pyroptosis by downregulating miR-143

**Figure 4.**
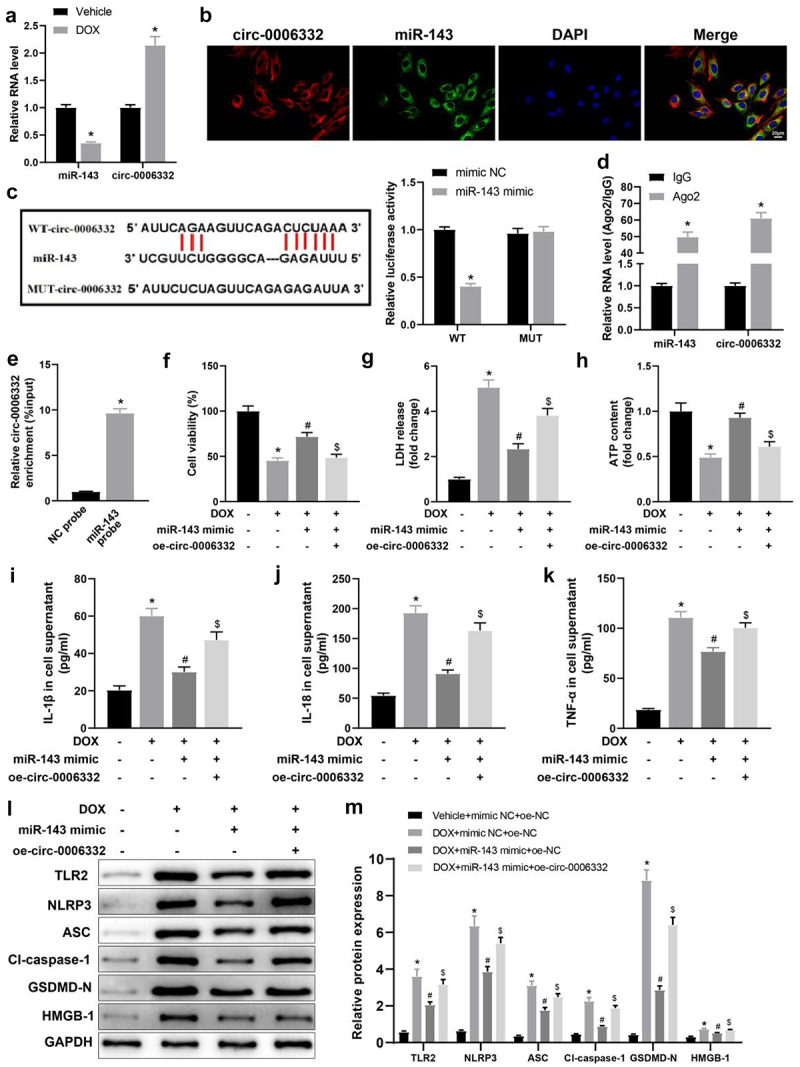
Circ -0,006,332 overexpression accelerates DOX-induced cardiomyocyte pyroptosis through miR-143 downregulation. a: RT-qPCR to detect miR-143 and circ -0,006,332 expression in rat heart tissues; b: the FISH assay to detect intracellular co-localization of circ -0,006,332 and miR-143 in cardiomyocytes; c: dual-luciferase assay to detect the binding relationship between miR-143 and circ -0,006,332 in H9C2 cells; d: circ 0006332 and miR-143 expression measured by RT-qPCR. e: RNA pull-down assay to detect the binding of miR-143 to circ -0,006,332; f: CCK-8 assay to detect cardiomyocyte viability; g: LDH release in cardiomyocyte supernatants; h: intracellular ATP contents; i-k: ELISA to detect IL-1β, IL-18, and TNF-α levels in cell supernatants; l-m: protein levels of TLR2 and pyroptosis-related molecules (ASC, cleave-caspase-1, GSDMD-N, NLRP3, and HMGB1) in cardiomyocytes detected by western blotting. In panel A, *p < 0.05 compared with the vehicle group; in panel C, *p < 0.05 compared with the WT + mimic NC group; in panel D, *p < 0.05 compared with the IgG group; in panels F-M, *p < 0.05 compared with the vehicle + mimic NC + oe-NC group, #*P* < 0.05 compared with the DOX + mimic NC + oe-NC group, and $ *p* < 0.05 compared with the DOX+ miR-143 mimic + oe-NC group. Values in the figures were measurement data and expressed as mean ± standard deviation. Two-group comparisons were analysed with the independent sample t-test (A, D, E), and one-way ANOVA with Tukey’s post hoc test (C-F-K, M) was used for comparisons among multiple groups. The cell experiments were repeated three times.

RT-qPCR data exhibited that miR-143 expression was plainly lower but circ -0,006,332 expression was evidently higher in rat myocardial tissues in the DOX group (vs. the Vehicle group) (Figure 4(a), *p* < 0.05). The FISH assay presented a significant co-localization of circ -0,006,332 and miR-143 in cardiomyocytes, illustrating an interaction between them (Figure 4(b)). As depicted in the results of the dual-luciferase assay, the luminescence intensity was reduced in the miR-143 mimic + WT group (vs. the mimic NC + WT group) but showed insignificant difference between the miR-143 mimic + MUT and mimic NC + MUT groups (Figure 4(c), *p* < 0.05). The RIP assay demonstrated that Ago2 bound to more miR-143 and circ -0,006,332 than IgG (Figure 4(d), *p* < 0.05). The RNA pull-down assay revealed that miR-143 probes recruited substantially more circ -0,006,332 than NC probes (Figure 4(e), *p* < 0.05). Altogether, miR-143 can directly bind to circ -0,006,332 in cardiomyocytes under DOX stimulation.

Subsequently, cell functions were detected. The data manifested that DOX treatment resulted in marked decreases in cell viability and ATP contents and a substantial enhancement in LDH release in cell supernatants, which were nullified by miR-143 mimic transfection. Moreover, further overexpression of circ -0,006,332 notably diminished cell viability and ATP contents and elevated LDH release in cell supernatants in DOX-treated cardiomyocytes in the presence of miR-143 mimic transfection (Figure 4f-h, *p* < 0.05).

Levels of IL-1β and IL-18 in cells supernatants and protein levels of TLR2 and pyroptosis-related molecules in cells were markedly augmented in cardiomyocytes by DOX treatment, which was abrogated by miR-143 mimic transfection. Additionally, overexpression of circ -0,006,332 strikingly annulled reductions in the levels of these factors caused by miR-143 mimic in DOX-treated cardiomyocytes (Figure 4i-m, *p* < 0.05).

In summary, circ -0,006,332 can inhibit miR-143 expression by directly binding to miR-143 and then facilitate DIC via TLR2 and the pyroptosis pathway.

### Overexpression of circ -0,006,332 reverses the alleviatory effect of miR-143 on DIC in rats

**Figure 5.**
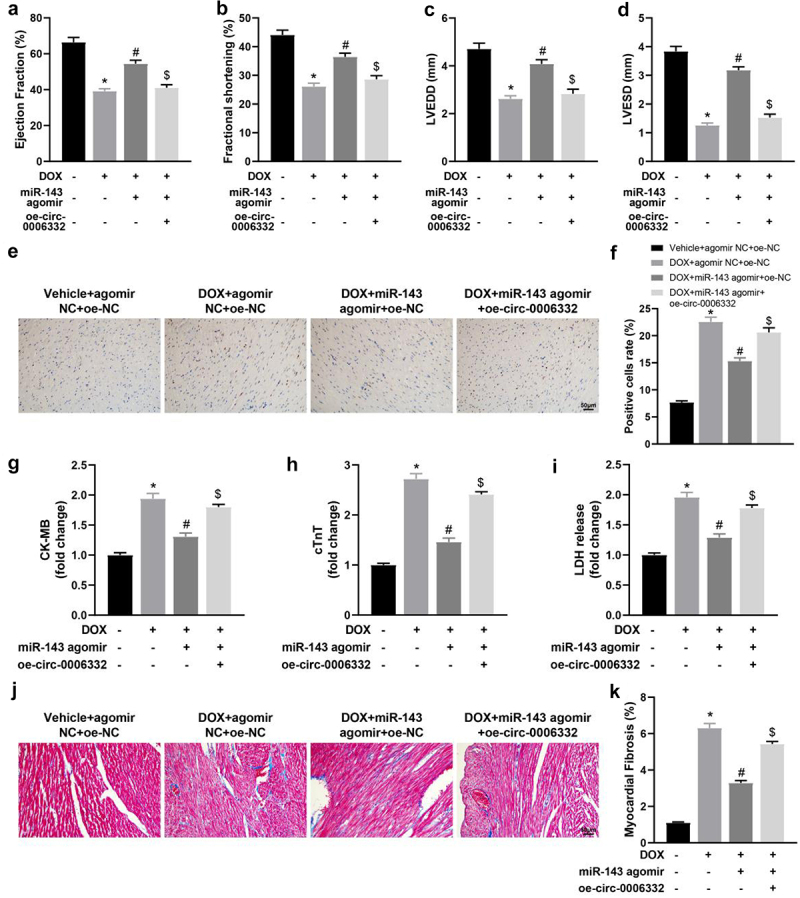
Overexpression of circ -0,006,332 reverses the improvement effect of miR-143 on DIC in rats. a-dd quantitative analysis of echocardiographic detection of cardiac function (LVEF, LVFS, LVEDD, and LVESD); e-f: TUNEL staining to detect apoptosis in myocardial tissues; g-i: ELISA to detect levels of CK-MB, cTnT, and LDH in myocardial tissues; j-k: Masson staining to observe myocardial tissue fibrosis, scale bar = 50 μm; *p < 0.05 compared with the vehicle + agomir NC + oe-NC group, #*P* < 0.05 compared with the DOX + agomir NC + oe-NC group, $*P* < 0.05 compared with the DOX + miR-143 agomir + oe-NC. Values in the figure were measurement data and expressed as mean ± standard deviation. One-way ANOVA with Tukey’s post hoc test (a-d, f-i, k) was used for comparisons among multiple groups.

Echocardiographic results revealed plainly decreased LVEF, LVFS, LVEDD, and LVESD in the DOX + agomir NC + oe-NC (vs. the Vehicle + agomir NC + oe-NC group) and DOX + miR-143 agomir + oe-circ -0,006,332 (vs. the DOX + miR-143 agomir + oe-NC) groups but increased in the DOX + miR-143 agomir + oe-NC group (vs. the DOX + agomir NC + oe-NC group) (Figure 5a-d, *p* < 0.05).

TUNEL staining results showed that the DOX + agomir NC + oe-NC and DOX + miR-143 agomir + oe-circ -0,006,332 groups had enhanced TUNEL-positive cells in rat myocardial tissues when compared with the Vehicle + agomir NC + oe-NC and DOX + miR-143 agomir + oe-NC group, respectively, which was contrary in the DOX + miR-143 agomir + oe-NC group versus the DOX + agomir NC + oe-NC group (Figure 5e,f, *p* < 0.05). ELISA results manifested increased serum levels of CK-MB, cTnT, and LDH in the DOX + agomir NC + oe-NC (vs. the Vehicle + agomir NC + oe-NC group) and DOX + miR-143 agomir + oe-circ -0,006,332 (vs. the DOX + miR-143 agomir + oe-NC) groups. On the contrary, these levels of these factors in the DOX + miR-143 agomir + oeNC group were notably reduced versus those in the DOX + agomir NC + oe-NC group (Figure 5g-i, *p* < 0.05).

Masson staining results demonstrated higher myocardial tissue fibrosis in the DOX + agomir NC + oe-NC group than in the Vehicle + agomir NC+ oe-NC group. Furthermore, fibrosis was alleviated in the DOX + miR-143 agomir + oe-NC group (vs. the DOX + agomir NC + oe-NC group) but enhanced in the DOX + miR-143 agomir + oe-circ -0,006,332 group (vs. the DOX + miR-143 agomir + oe-NC group) (Figure 5j,k, *p* < 0.05).

### Overexpression of circ -0,006,332 neutralizes the inhibition of miR-143 upregulation on DOX-stimulated myocardial pyroptosis in rats

**Figure 6.**
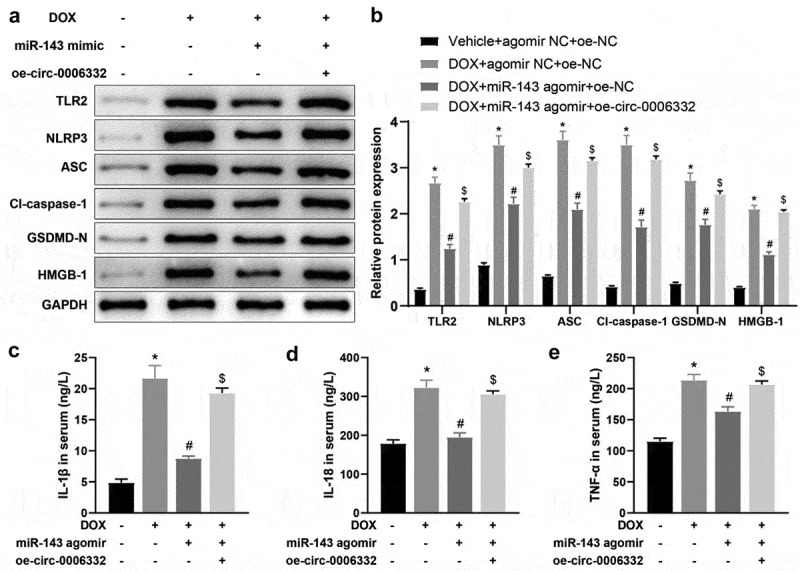
Overexpression of circ -0,006,332 nullifies the inhibition of miR-143 on DOX-induced cardiomyocyte pyroptosis in rats. a-b: Western blotting to detect levels of TLR2 protein and pyroptosis-related proteins (ASC, cleave-caspase-1, GSDMD-N, NLRP3, and HMGB1) in cardiac tissues; c-e: ELISA to detect IL-1β, IL-18, and TNF-α in rat serum; *p < 0.05 compared with the vehicle + agomir NC + oe-NC group, #*P* < 0.05 compared with the DOX + agomir NC + oe-NC group, $*P* < 0.05 compared with the DOX + miR-143 agomir + oe-NC group. Values in the figure were measurement data and expressed as mean ± standard deviation. One-way ANOVA and Tukey’s post hoc test were used for comparison among multiple groups (b-e).

Further validation was performed at the animal level. TLR2, NLRP3, ASC, cleave-caspase-1, GSDMD-N, and HMGB1 levels in the heart tissues were remarkably increased in the DOX + agomir NC + oe-NC group (vs. the Vehicle + agomir NC + oe-NC group), lower in the DOX + miR-143 agomir + oe-NC group (vs. the DOX + agomir NC + oe-NC group), and enhanced in the DOX + miR-143 agomir + oe-circ -0,006,332 group (vs. the DOX + miR-143 agomir + oe-NC group) (Figure 6a,b, *p* < 0.05).

ELISA results manifested higher serum levels of IL-1β, IL-18 and TNF-α in the DOX + agomir NC + oe-NC (vs. the Vehicle + agomir NC + oe-NC group) and DOX + miR-143 agomir + oe-circ -0,006,332 (vs. the DOX + miR-143 agomir + oe-NC) groups but lower levels in the DOX + miR-143 agomir + oe-NC group (vs. the DOX + agomir NC + oe-NC group) (Figure 6c-e, *p* < 0.05).

**Figure 7.**
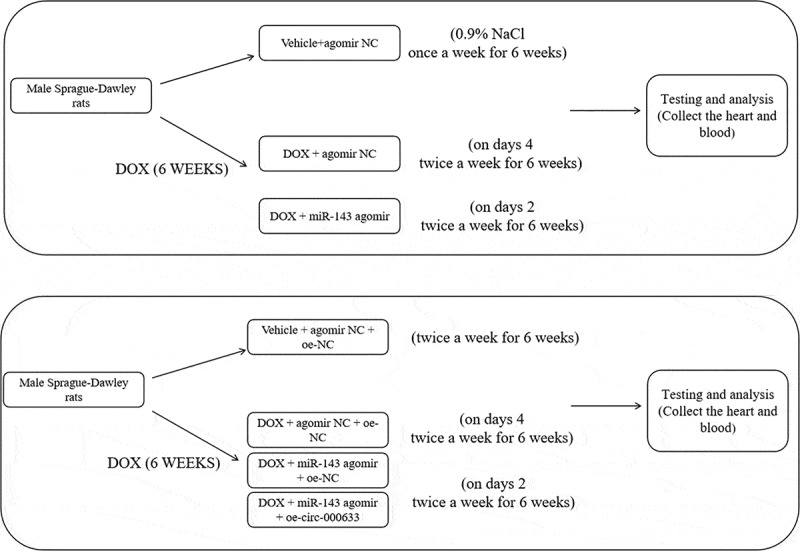
The schematic diagram in the rat experimental protocol.

Collectively, circ -0,006,332 upregulation depresses pyroptosis in rat myocardial tissues through the miR-143/TLR2 axis and thus participates in DOX-induced cardiac injury.

## Discussion

Due to their stability, widespread distribution, and high sensitivity in human body, miRNAs can be used as biomarkers for DIC, as confirmed by several studies in DIC cell/animal models or DIC patients [15]. Many circRNAs have been recognized to participate in DIC through the miR/mRNA axis. For example, circ_0001312 reverses the cytotoxic effects of DOX on cardiomyocytes through the miR-409-3p/HMGB1 axis [21]. Circ-LTBP1 enhances DOX-caused intracellular toxicity in cardiomyocytes through the miR-107/ADCY1 axis [22]. Accordingly, our study explored the circRNAs/miR/mRNA axis implicated in DIC. Our experimental results delineated that circ -0,006,332 promoted cardiomyocyte pyroptosis by binding to miR-143 and upregulating TLR2, thus increasing DOX-induced cardiac damage.

Myocardial injury is mainly reflected by the levels of CK, CK-MB, LDH, and cTnT, and all of these enzymes can be elevated by DOX in mice [39]. As a marker of mitochondrial function, lower ATP activities indicate mitochondrial dysfunction, and DOX markedly augments LDH and CK-MB levels and diminished ATP levels in mitochondrial homogenate [40]. DOX also induced cardiomyocyte pyroptosis, impaired cardiac function, and caused myocardial injury in rats and mice [41,42]. DOX administration could elevate the expression of pyroptotic markers and pro-inflammatory cytokines in H9C2 cells [43] and contribute to cardiotoxicity [44]. Similarly, our data unravelled that DOX reduced cell viability and ATP levels but enhanced LDH release, IL-18, IL-1β, and TNF-α levels, and the expression of pyroptosis-related molecules (NLRP3, ASC, cleave-caspase-1, GSDMD-N, and HMGB1) in cardiomyocytes, as well as elevating cardiac dysfunction, apoptosis, CK-MB, cTnT, and LDH levels, and fibrosis in myocardial tissues of rats.

In addition, our results indicated that DOX caused a decrease in miR-143 expression in cardiomyocytes and that miR-143 overexpression could relieve DOX-stimulated pyroptosis *in vivo* and *in vitro* and depress myocardial fibrosis, cardiac dysfunction, and myocardial injury caused by DOX in rats. Reportedly, increased plasma miR-143 levels may suggest recovery of cardiac function at the chronic phase in patients with acute myocardial infarction [45]. Many researchers have reported the repressive role of miR-143 in pyroptotic and pro-inflammatory markers. For instance, miR-143 overexpression diminishes NLRP3, cleaved-caspase-1, IL-1β, and IL-18 levels in orbital fibroblasts [46]. miR-143 depletion elevates pyroptotic markers in primary microglia [47]. As for fibrosis, circ_DLGAP4 facilitates mesangial cell fibrosis, which was nullified by miR-143 upregulation [48]. Likewise, miR-143-5p can hinder renal fibrosis progression in mice [49]. miR-143 is required for the attenuating effects of 6-Gingerol on hypoxia/reoxygenation-induced cytotoxicity and cardiomyocyte injury [50]. These findings and evidence plausibly support the protective role of miR-143 against DIC.

Then, this study focused on the upstream and downstream mechanism of miR-143 in DIC. Previous research found miR-143 could specifically bind to circ_0006332 [24]. Another study demonstrated that miR-143 could inhibit its potential target gene TLR2 in hepatoma [38]. Consistently, our results confirmed that circ_0006332 bound to miR-143 to mediate TLR2 expression. Of note, prior studies revealed that in vivo DOX treatment increased TLR2 levels in cardiomyocytes [38] and inhibition of TLR2 activity improved cardiac dysfunction and inhibited cardiac fibrosis and myocardial inflammation [51]. TLR2 downregulation is reported to be involved in the repressive effect of 8-OHdG treatment on DOX-induced pyroptosis in cardiomyocytes [14]. Additionally, existing evidence on circ_0006332 is scare, so we centred on it in subsequent experiments. As our data indicated, circ -0,006,332 was notably increased in DOX-induced cardiomyocytes, and circ -0,006,332 overexpression reduced cell viability and ATP contents, elevated LDH release, pyroptosis, and inflammation in cardiomyocytes in the presence of miR-143 mimic. Moreover, circ -0,006,332 overexpression caused cardiac dysfunction, increased CK-MB, cTnT, and LDH levels, and enhanced fibrosis and pyroptosis in DOX-treated rats in the presence of miR-143 agomir. Therefore, we suspect that circ_0006332 May participate in DIC through the miRNA-143/TLR2 axis. Although there is currently no research on the role of circ -0,006,332 in DIC and related fields, there are numerous studies on other circRNAs. For instance, circ-SKA3 silencing accelerated cell viability and prevented cell apoptosis in DOX-treated AC16 cells, reducing DIC through the miR-1303/TLR4 axis [52]. CircRNA DICAR deficiency could induce cardiac dysfunction and fibrosis in mice [53]. CircHelz activates NLRP3 inflammasomes to induce inflammation, pyroptosis, and myocardial injury by binding to miR-133a-3p in ischaemic mouse heart [54]. Overall, our results and prior findings hinted that overexpression of circ -0,006,332 increased cardiomyocyte pyroptosis to facilitate DIC.

In conclusion, this article experimentally determined that circ -0,006,332 specifically bound to miR-143 and then upregulated TLR2 expression and primarily suggested the impacts of this circRNA/miRNA/mRNA network on pyroptosis in DIC, showing their promise as treatment targets for DIC. However, mitochondrial metabolism, autophagy, and oxidative stress are also implicated in DIC. Further experiments are necessary to comprehensively explore this circRNA/miRNA/mRNA network in the above aspects and further study the downstream pathways.
